# Microencapsulation of Red Grape Juice by Freeze Drying and Application in Jelly Formulation

**DOI:** 10.17113/ftb.58.01.20.6429

**Published:** 2020-03

**Authors:** Liliana Mihalcea, Vasilica Barbu, Elena Enachi, Doina Georgeta Andronoiu, Gabriela Râpeanu, Maricica Stoica, Loredana Dumitrașcu, Nicoleta Stănciuc

**Affiliations:** Integrated Center for Research, Expertise and Technological Transfer in Food Industry, Faculty of Food Science and Engineering, Dunarea de Jos University of Galati, 111 Domnească Street, 800201 Galați, Romania

**Keywords:** anthocyanin microencapsulation, freeze drying, whey protein isolate-chitosan matrix, grape juice, jelly, *in vitro* digestibility

## Abstract

The aim of this work is to obtain new food products enriched with bioactive compounds from concentrated grape juice microencapsulated by freeze drying using a whey protein isolate-chitosan system. The obtained powder showed an encapsulation efficiency of (86.1±4.0) %, with an anthocyanin mass fraction (expressed as cyanidin-3-*O*-glucoside equivalent) of (1.4±0.2) mg/g, while the total polyphenolic (expressed as gallic acid equivalents) and flavonoid (expressed as catechin equivalents) mass fractions were (3.3±0.6) and (1.6±0.5) mg/g, respectively. The confocal laser microscopy revealed the presence of the flavonoid pigments wrapped inside the matrix, whereas the anthocyanins were grouped into large and compact clusters. The microencapsulated powder was used for jelly formulation. The new food formulations have a satisfactory anthocyanin mass fraction ranging from (0.03±0.01) to (0.12±0.02) mg/g, while no significant differences were observed in flavonoid content. All the value-added jelly showed appreciable antioxidant activity. The *in vitro* digestibility results confirm a slow release of anthocyanins from the food matrices during simulated gastric digestion and a significant release of the bioactive compounds into the gut. The addition of microencapsulated powder caused a significant decrease in firmness, cohesiveness and springiness, leading to the destabilization of the gel structure, while reducing the attraction forces between the matrix components. The sensorial analysis indicated that the panellists preferred the sample with grape juice the most.

## INTRODUCTION

New alternatives to synthetic colourants in food applications are increasing due to the consumer demands for natural pigments. The grapes are suitable source of bioactive compounds and pigments. Grapes are consumed in different states, such as fresh, processed in the winemaking, jam, jelly, juice, vinegar, extract and oil production. The wine industry generates large amounts of by-products, *e.g*. skins, seeds and stems, rich in bioactive compounds such as polyphenols (*1*). The grape production in Romania in 2015 was 799 kt (*2*) and almost all grape pomace was used as fertilizer, animal feed or for vinegar production. Unfortunately, the use of grape pomace for agricultural purposes is limited due to its high content of polyphenols including flavonoids, phenolic acids, stilbenes and tannins that are potentially toxic to the soil microbiota (*3*). Meini *et al.* (*4*) suggest that, nowadays, the main potential uses of grape pomace include the extraction of tartaric acid, ethanol production, distillation processes, as fertilizer and as an additive in animal feed. However, the applicability of grape pomace is limited due to the fact that at high mass fractions of phenolics the germination is inhibited, whereas the presence of polymeric polyphenols reduces the digestibility.

Grape pomace is considered a valuable by-product because it contains bioactive compounds such as polyphenols (*5*-*7*), anthocyanins and flavonols (*8*), resveratrol (*9*) and dietary fibre with significant benefits for human health due to their anti-apoptotic, anti-cancer, antioxidant, antibacterial and anti-hypercholesterolemic activities (*10*-*13*). The development of bioactive products for food and pharmaceutical industries might add value to and increase industrial applicability of grape pomace (*14*). Alternatively, the grape pomace may be considered a good source for natural bright ruby red pigments. However, the applications of the polyphenols and anthocyanins as natural colourants in foods at industrial level are limited by their low stability during processing and storage (*15*, *16*).

In this context, microencapsulation is an efficient technique to increase the stability and protection of bioactive compounds from the environmental conditions. Moreover, the microencapsulation involves a coating process, the isolation and the controlled release of bioactive compounds in a new functional food formula. Many methods have been used for the encapsulation of anthocyanins: spray drying of barberry anthocyanins in combinations with gum Arabic, maltodextrin and gelatine (*17*, *18*), phenolic compounds from Violeta red grape cultivar encapsulated in soy protein, whey protein and maltodextrin (*19*), resveratrol encapsulated in β-glucans and soy lecithin with particles from gas-saturated solutions (PGSS) drying method (*20*), and encapsulation of anthocyanins from sour cherries using β-lactoglobulin by freeze drying (*21*).

Therefore, the aim of the present study is to microencapsulate the Băbească neagră red grape juice obtained from the sweet pomace in whey protein isolate and chitosan by freeze drying. Encapsulation efficiency, polyphenolic, anthocyanin and flavonoid contents, and antioxidant activity of the obtained microcapsules were characterized. Microstructures of the particles were observed by confocal scanning laser microscopy. In order to demonstrate the functional properties of the powder as potential food ingredient, two types of jelly were obtained in which the functionality of the powder was tested as natural food colourant and antioxidant. The antioxidant activity, polyphenolic, anthocyanins and flavonoid contents, physicochemical properties, *in vitro* digestibility, textural and sensorial analysis of the jelly were investigated.

## MATERIALS AND METHODS

### Materials and chemicals

Whey protein isolate (protein content 90%) was purchased from Fonterra (Clandeboye, New Zealand). 2,2-Diphenyl-1-picrylhydrazyl (DPPH), 6-hydroxy-2,5,7,8-tetramethylchromane-2-carboxylic acid (Trolox), aluminium chloride, Folin-Ciocalteu reagent, sodium hydroxide, KH_2_PO_4_, gallic acid, catechin, Congo Red, 4',6-diamidine-2'-phenylindole dihydrochloride (DAPI), pepsin, pancreatin and ethanol (HPLC grade) were obtained from Sigma-Aldrich, Merck, Steinheim, Germany. For jelly preparation, commercial grade agar-agar (Naturtrade, Szeged, Hungary), stevia (Gerble from Nutrivita, Ilfov, Romania) and citric acid were purchased from the local supermarket (Galați, Romania). The sweet pomace of the Băbească neagră red grapes was provided from The Research and Development Center for Grape Growing and Wine Production, Târgu Bujor, Galati, Romania, in September 2017.

### Preparation of juice from the red grape pomace

The Băbească neagră pomace was pressed using a juicer with maximum power 900 W (model AE 3666 Automatic juicer; Clatronic Int. GmbH, Kempen, Germany). After pressing, the juice was collected, filtered through a double filter and concentrated by using a rising film evaporator FT22-A (Armfield, Ringwood, Hampshire, UK) with the following parameters: steam pressure 3 MPa, product flow 8 L/h and temperature 94.3 °C. After concentration, the concentrated juice was filled in plastic bottles and stored at 4 °C until analysis.

### Determination of pH, total soluble solids and moisture content

To determine the pH of the concentrated juice, a portable pH-meter (model s20-k; Mettler Toledo, Columbus, OH, USA) was used. Total soluble solid content was determined with an Abbe refractometer (model 320; PCE Instruments, Southampton, Hampshire, UK), and results were expressed in °Brix.

### Preparation of the microencapsulated powders

A modified method described by da Silva Bastos *et al.* (*22*) was used to encapsulate concentrated juice in whey protein isolate (WPI) and chitosan. In brief, powdered WPI (12% *m*/*V*) and chitosan (1.5%) were dissolved in 10 mM Tris buffer (pH=6.0), and stirred at ambient temperature for at least 2 h to ensure complete hydration. The WPI/chitosan ratio was 1:0.02. The concentrated juice (45 mL) was homogenized with chitosan solution using a blender, under stirring at full speed for a few seconds, followed by the addition of WPI solution to the mixture by gentle hand movements. The mixture was freeze dried (Alpha 1-4 LD plus; CHRIST, Osterode am Harz, Germany) at -42 °C under the pressure of 10 Pa for 48 h. Afterwards, the powders were collected and packed in metallized bags, and kept in a freezer at -20 °C until analysis.

### Encapsulation efficiency

The method involves the measurement of total anthocyanin (TAC) and surface anthocyanin (SAC) content in the microencapsulated powder, as described by Oancea *et al.* (*21*). Quantification was carried out using the pH differential method described by AOAC Official Method 2005.02 (*23*). The method is based on the colour change of the monomeric anthocyanin pigments depending on the pH. The absorbance difference of the pigments measured at 520 nm is proportional to the pigment concentration. Encapsulation efficiency (EE/%) was calculated according to the following equation:

 /1/

### Quantification of total polyphenols and total flavonoids in concentrated juice and microencapsulated powder

The phytochemical content of the powder was determined as described by Stănciuc *et al.* (*24*). In brief, the total polyphenolic content (TPC) was determined using the Folin-Ciocalteu method (*25*), and the results were expressed in mg gallic acid equivalents (GAE) per g dry powder. The method is based on the electron transfer from phenolic compounds to Folin-Ciocalteu reagent, in an alkaline solution, to form a blue chromophore with the maximum absorption at *λ*=765 nm. The intensity of the colour was measured with UV-Vis double-beam spectrophotometer with data analysis software (Jenway Scientific Instruments, Essex, UK).

The total flavonoid content (TFC) was determined using a colourimetric method (*26*). To 0.25 mL of the solutions obtained from total anthocyanin (TAC) analysis, sodium nitrite (5%), aluminium chloride (10%) and sodium hydroxide (1 M) were added. The principle of the method involves the formation of acid-stable complexes of aluminium chloride with flavones and flavonols, while the absorbance was measured at 510 nm against the suitable blank. The TFC was expressed in mg catechin equivalents (CE) per g of dry powder.

### Antioxidant activity

The antioxidant capacity was determined as described by Yuan *et al.* (*26*) by measuring DPPH radical scavenging activity, expressed as Trolox equivalent (TE) in mmol per g of dry powder using a calibration curve. This method is based on the reduction of stable DPPH nitrogen radicals in the presence of antioxidants and the change in the absorbance was measured spectrophotometrically (Jenway Scientific Instruments) at 515 nm.

### Confocal laser scanning microscopy

Confocal microscopy studies were performed by using LSM 710 inverted confocal microscope connected to a Zeiss Axio Observer Z1 inverted microscope (Carl Zeiss X-ray Microscopy, Inc., Pleasanton, CA, USA). The laser scanning system is composed of several lasers such as diode laser (405 nm), an Ar laser (458, 488 and 514 nm), a diode-pumped solid-state (DPSS) (561 nm), and a HeNe-laser (633 nm). The microencapsulated powder both native and stained with fluorescent dyes Congo Red (40 µM) and 4′,6-diamidino-2-phenylindole (DAPI) (1 µg/mL) (ratio 3:1:1, *m*/*V*/*V*) was analysed. In order to observe the distribution of the flavonoid pigments into the WPI and chitosan matrix, the FS15, FS38 and FS49 filters were used while the ZEN 2012 SP1 software (Black Edition) (*27*) was used for the image analysis.

### Application of microencapsulated powder in jelly formulation

A comparative study of the use of concentrated juice and microencapsulated grape juice powder as natural sources of bioactive compounds in jelly formulation was performed. Agar-agar was used as superior gelling agent that remains solid at 37 °C, characterized by stability, clarity, metabolic inertness and nontoxicity (*28*). Four jelly formulations were considered: a control sample without any addition of bioactive compounds (C), jelly with 10% concentrated juice (S), jelly with 5% (S1) and 10% microencapsulated grape juice (S2), respectively.

The procedure consists of a first step of mixing the ultrapure water with concentrated juice or microencapsulated powder and agar-agar, stevia and citric acid under continuous stirring to avoid the formation of lumps. The final mixture was then boiled for 3 min and filled in the silicone forms with different geometric shapes. The jelly samples were refrigerated at 4 °C.

### Physicochemical analysis of jelly

Physicochemical characteristics (moisture content, total sugar, ash, fat, protein and energy value) of the value-added and control samples of the jelly were analysed according to standardized and validated laboratory methods, such as the gravimetric methods for determining the moisture, sugar and ash contents from sweet products (*29*-*31*), the Kjeldahl method for the protein (*32*) and gravimetric method for fat content (*33*).

### In vitro digestibility

The procedure for *in vitro* digestibility was a modification of that described by Peixoto *et al.* (*34*) and Oancea *et al*. (*21*). The method involves a two-step digestion pattern evaluation: the first one in simulated gastric fluid, which consists of porcine pepsin (40 mg/mL in 0.1 M HCl) for 2 h and the second one in simulated intestinal fluid consisting of pancreatin (2 mg/mL) in KH_2_PO_4_ (0.05 M) at pH=7.5. The pH of the system was adjusted to 7.0 with 0.1 M NaOH, prior to the incubation of the samples for 2 h. The incubation was performed in an SI–300R orbital shaking incubator (Medline Scientific, Chalgrove, UK) at 100 rpm and 37 °C. The total anthocyanin content of the samples was measured every 30 min during the *in vitro* digestion.

### Textural parameters

The textural parameters (firmness, cohesiveness and springiness) were determined by the texture profile analysis method using the Brookfield CT3 Texture Analyzer (AMETEK Brookfield, Middleborough, MA, USA). The jelly samples were cut in cylindrical pieces with 12 mm length and 8 mm diameter. A double compression, without holding time, was applied using an acrylic cylinder probe (TA11/1000) with 24.5 mm diameter and 35 mm height. The load cell was 1000 g and the trigger load 0.067 N. The compression distance was 4 mm, pre-test, test and post-test speeds were 2 mm/s. The data were recorded and processed using the TexturePro CT v. 1.5 software (*35*). For each sample, five tests were performed.

### Sensory analysis

A panel consisting of six panellists aged between 29 and 40, 3 male and 3 female, evaluated the sensorial characteristics of jelly according to a five-point hedonic scale. The panellists evaluated the colour (colourless to burgundy), aroma associated with concentrated juice (weak to strong), roughness (soft to rough), elasticity (low to very high), moisture (low to very high), hardness (low to very hard), sweetness (weak to strong), and overall likeness of the product (dislike to like extremely). Samples were coded and then served randomly to the panellists on white papers. Water was used for mouth rising before and between samples.

### Statistical analysis of the data

Unless otherwise stated, the data reported in this study represent the average values of triplicate analyses and reported as mean value±standard error of the mean. The analysis of variance (ANOVA, p<0.05) was carried out to assess significant differences between the values. Statistical evaluation of the sensorial results was performed using Minitab 18 statistical processing software (*36*). First, the data were checked for normality using Ryan-Joiner test, which is similar to Shapiro-Wilk test, and equality of variances using Bartlett test. Then, one-way ANOVA was used to identify if panellists detected any differences between samples considering a significance level of p<0.05. Dunnett’s multiple comparison test with a control was employed when appropriate.

## RESULTS AND DISCUSSION

### Physicochemical properties of the grape juice

The physicochemical properties of concentrated juice are influenced by the concentration parameters and by the grape cultivar. In our study, the Băbească neagră juice was concentrated at temperature of 94.3 °C and steam pressure of 3 MPa for 500 min. After concentration, the physicochemical properties were pH=4.11 and soluble solid content 43.5 °Brix. Moser *et al.* (*19*) reported the values of pH=3.86 and 14 °Brix for the Victoria red grape cultivar juice after steam extraction at 75-85 °C for 60 min.

### Encapsulation efficiency and phytochemical parameters of microencapsulated powder

After microencapsulation of concentrated juice, the resulting powder was analyzed with respect to EE, TAC, TPC, TFC and antioxidant activity. The EE was (86.1±4.0) %. Higher values of (99.65±0.08) and (94.10±0.61) % of EE were reported by Stănciuc *et al.* (*24*) for anthocyanins extracted from grape peels and encapsulated in WPI and acacia gum and pectin, respectively. Oancea *et al.* (*21*) encapsulated anthocyanin extract from sour cherry skins in β-lactoglobulin in its native, heat-treated and cross-linked states and reported lower EE values of (54.14±0.67), (44.79±0.90) and (64.69±0.24) %, respectively. Akhavan Mahdavi *et al.* (*17*) encapsulated anthocyanins from barberry (*Berberis vulgaris*) extract in a combination of gum Arabic and maltodextrin, maltodextrin and gelatin and maltodextrin alone by spray drying. These authors reported that for all four ways of EE was high and approx. 92-93%.

The powder contained on dry mass bases TAC expressed as cyanidin-3-*O*-glucoside equivalent (CGE) of (1.4±0.2) mg/g, whereas the TPC (as GAE) and TFC (as CE) were (3.3±0.6) and (1.6±0.5) mg/g, respectively. Oancea *et al.* (*21*) reported significantly lower value for TAC extracted from sour cherry skins and encapsulated in β-lactoglobulin in different conformational states, ranging from 0.13 to 0.14 g/g. Stănciuc *et al.* (*24*) reported values of (0.43±0.05) mg/g for TPC and (3.07±0.11) mg/g for the anthocyanins extracted from grape peels and encapsulated in WPI and acacia gum and pectin. They suggested that a higher content of flavonoids was entrapped in the variant with pectin, (0.45±0.09) mg/g, than in the variant with acacia gum, (0.22±0.03) mg/g. However, TAC was significantly lower, (0.014±0.0005) and (0.018±0.001) mg/g, respectively.

### Morphological properties of the powders

**Fig. 1:**
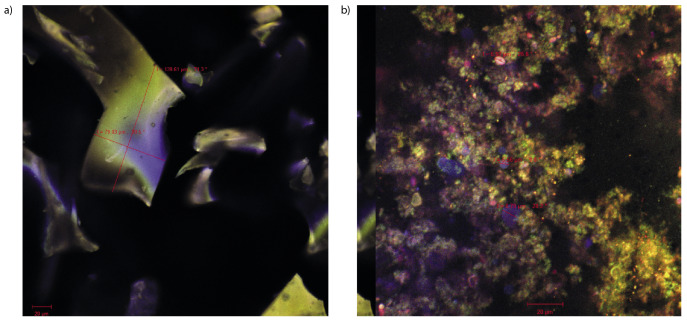
Confocal laser scanning microscopy images of the microencapsulated grape juice powder obtained with a 40× objective: a) native and b) fluorochrome-stained complex

A confocal micrograph of the microencapsulated powder without any staining is shown in Fig. 1a. Due to autofluorescence phenomenon, it could be observed that the native powder formed numerous irregular and thin scales with variable sizes between 79.03 and 139.61 µm. By microencapsulating, the pigments were wrapped inside the matrix formed between the whey proteins and linear polysaccharides of chitosan. The anthocyanins were grouped into large and compact clusters that can be observed in Fig. 1b. The bioactive compounds (the small green spherosomes) were distributed between some protein fractions (stained with Congo Red dye shown in yellow or red) or between some chitosan spherosomes (5-10 µm, in blue).

### Formulation and characterization of jelly products

The use of natural colourants in processed foods and beverages is important for increasing consumer acceptability, and also improving their potential health benefits (*37*). In our study, the concentrated and microencapsulated concentrated juices were used to formulate three different variants of jelly, at concentration of 10% concentrated juice (S), and 5% (S1) and 10% microencapsulated concentrated juice (S2), in order to evaluate the content of phytochemicals and texture, together with acceptable sensory attributes and physicochemical evaluations.

Therefore, TAC, TPC, TFC and antioxidant activity of the value-added jelly were characterized (Table 1). As expected, the highest TAC (as CGE) and TPC (as GAE) mass fractions on dry mass basis of (0.12±0.02) and (0.09±0.01) mg/g, respectively, were found in S2, whereas no significant differences were observed in TFC content between the samples (Table 1). Surprisingly, the highest antioxidant activity expressed as TE on dry mass basis of (0.9±0.1) mmol/g was found in S, probably due to the higher content of phytochemicals. However, it may be appreciated that all the value-added jelly showed appreciable antioxidant activity.

**Table 1:** Physicochemical and phytochemical characteristics of value-added jelly

Property	C	S	S1	S2
*w*(NaCl)/% of which *w*(Na)/%	0.080±0.004 0.032±0.001	0.080±0.005 0.030±0.001	0.080±0.006 0.032±0.001	0.080±0.004 0.030±0.001
*w*(ash)/%	0.120±0.006	0.130±0.001	0.150±0.001	0.180±0.002
*w*(moisture)/%	85.6±2.6	84.6±0.6	81.8±1.1	78.7±1.1
*w*(fat)/%	0	0	0	0
*w*(protein)/%	0	0.100±0.001	1.200±0.001	2.10±0.04
*w*(carbohydrates)/%	14.3±0.6	15.2±0.3	16.8±0.9	19.0±0.8
*E*/kcal *E*/kJ	58.54 244.70	62.60 261.66	74.00 309.32	86.59 361.98
*w*(TPC as GAE)/(mg/g)	0	0.020±0.004	0.07±0.01	0.09±0.01
*w*(TFC as CE)/(mg/g)	0	0.070±0.005	0.070±0.004	0.074±0.004
*w*(TACas CGE)/(mg/g)	0	0.03±0.01	0.07±0.01	0.12±0.02
*w*(antioxidant activity as TE)/(mM/g)	0	0.9±0.1	0.87±0.01	0.82±0.01

Results are expressed as mean value±standard deviation. C=control, S=jelly with the addition of grape juice (10%), S1 and S2=jelly with the addition of microencapsulated powder 5 and 10%, respectively, TPC=total polyphenolic content, TFC=total flavonoid content, TAC=total anthocyanin content, GAE=gallic acid equivelents, CE=catechin equivalents, CGE=cyainidin-3-glucoside equivalents, TE=Trolox equivalents

The formulated jelly had lower moisture content ((84.6±0.6), (81.8±1.1) and (78.7±1.1) %, respectively) than control jelly (85.6±2.6) % (Table 1). The protein content in value-added jelly were (1.20±0.001) % in S1 and (2.10±0.04) % in S2, higher than in control and S, which can be attributed to the presence of WPI in the microencapsulated powders. However, the calculated energy values were higher of value-added jelly (62.60, 74.00 and 86.59 kcal/100 g, respectively) than of control (58.54 kcal/100 g).

### In vitro digestibility of the anthocyanins in the jelly

**Fig. 2:**
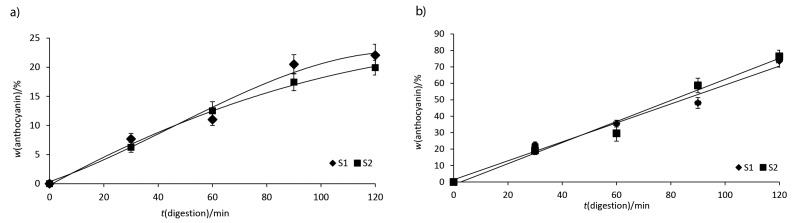
Release of anthocyanins from formulated jelly during digestion in: a) simulated gastric juice and b) simulated duodenal juice. S1=samples with the addition of 5% microencapsulated grape juice, S2=samples with the addition of 10% microencapsulated grape juice

Simulated digestion was applied to determine the amount of anthocyanins that were available for uptake and transport in order to be bioavailable (*38*). The digestion pattern of formulated jelly is given in Fig. 2. Fig. 2a shows that the anthocyanin release was limited during *in vitro* simulated gastric digestion, suggesting that whey proteins played a significant role in protecting biologically active compounds, thus allowing a possible release into the gut, as suggested by Shpigelman *et al.* (*39*).

As it can be seen from Fig. 2a, the digestion of anthocyanins (maximum release approx. 23%) was moderate S1, and maximum release of approx. 20% after 120 min of reaction in S2. From our results, it seems that more than 77% of the encapsulated anthocyanins in S1 and 80% in S2 were retained in the formulated jelly during *in vitro* gastric digestion. Therefore, it can be concluded that anthocyanins were slowly released from the jelly under simulated digestion conditions. Sari *et al.* (*40*) reported similar results suggesting that curcumin is released slowly from the nanoemulsion under simulated digestion conditions.

Fig. 2b shows the duodenal digestion of the products. S2 samples obtained after 120 min of peptic digestion were hydrolyzed faster by pancreatin than S1 samples. However, a significant release of anthocyanins in the duodenal simulated juice can be observed, up to 74% in the case of S1 and 76% in the case of S2, respectively.

To conclude, our *in vitro* digestibility results support a slow release of anthocyanins from the food matrices during simulated gastric digestion and a significant release of the bioactive compounds into the gut.

### Textural analysis of the jelly

The textural parameters of jelly are presented in Table 2. Control and the sample with added concentrated juice had the highest firmness results of (10.41±0.40) and (2.68±0.15) N, respectively (Table 2). The increase in the addition of microencapsulated grape juice caused a significant decrease in firmness from (5.5±0.3) to (278±0.2) N of S2 and S1, respectively. A similar tendency was noticed for the cohesiveness and springiness, concluding that the addition of microencapsulated grape juice leads to destabilization of the gel structure, reducing the attraction forces between the matrix components and the ability to recover the deformation. The results are in contradiction with those reported by Akhavan Mahdavi *et al.* (*17*) for jelly with encapsulated barberry anthocyanins, who did not observe any significant differences in texture parameters.

**Table 2:** Textural parameters of added values jelly determined by texture profile analysis

Property	C	S	S1	S2
Firmness/N	10.4±0.4	2.7±0.2	10.4±0.4	5.5±0.3
Cohesiveness	0.6±0.1	0.28±0.07	0.5±0.1	0.3±0.0
Springiness/mm	1.3±0.1	0.89±0.07	1.2±0.2	1.0±0.1

Results are expressed as mean of five determinations±standard deviation. C=control sample, S=jelly with the addition of grape juice (10%), S1 and S2=jelly with the addition of microencapsulated powder of 5 and 10%, respectively

### Sensory analysis of the jelly

Table 3 shows the average scores of sensorial attributes evaluated by the panellists. Control sample, as expected, had the lightest colour, while S had the darkest (p<0.001). Regarding the aroma associated with grape juice, S sample was perceived by the panellists as the sample that had the strongest aroma of grape juice. Roughness and moisture of all samples were evaluated as being similar (p>0.05). Scores given for elasticity varied among samples, the highest elasticity being attributed to S2. Panellists evaluated S2 as the sample that has the lowest hardness (p<0.05). No differences in the sweetness of the samples were detected by the panellists. Overall acceptability indicated sample S as being the most preferred by the panellists.

**Table 3:** Sensory characteristics of jelly

Sensorial attribute	C	S	S1	S2	p-value
Colour	(1.0±0.0)^a^	3.8±0.6	1.9±0.2	3.1±0.2	0.000
Aroma associated with grape juice	(1.0±0.0)^a^	2.8±0.8	2.0±0.6	2.2±0.8	0.001
Roughness	1.0±0.0	1.0±0.0	1.3±0.8	1.5±0.5	>0.05
Elasticity	1.3±0.8	1.8±0.8	1.7±1.0	2.7±1.2	>0.05
Moisture	2.0±0.9	1.5±0.5	1.8±0.8	2.3±1.2	>0.05
Hardness	(3.4±0.5)^a^	(3.5±0.5)^a^	(3.3±0.8)^a^	2.5±0.5	<0.05
Sweetness	2.3±0.8	2.5±0.5	1.8±0.8	1.7±0.5	>0.05
Overall acceptability	(2.8±0.8)^a^	3.5±0.5	3.2±1.2	(2.3±1.0)^a^	<0.05

Mean values without a letter in superscript are significantly different from the control based on Dunnett’s multiple comparison test with a control. C=control sample, S=jelly with grape juice (10%), S1 and S2**=**jelly with the addition of microencapsulated grape juice 5 and 10%, respectively

## CONCLUSIONS

In this study, the phytochemicals from concentrated red grape juice were successfully microencapsulated in a whey protein isolate-chitosan system, with encapsulation efficiency of anthocyanins of 86%. The composition of phytochemicals and particle morphology of the powder were characterized. The confocal laser microscopy revealed the presence of flavonoid pigments wrapped inside the matrix, with anthocyanins grouped into large and compact clusters. Value-added food products were obtained by the addition of the concentrated and microencapsulated grape juice to jelly recipes. The jelly presented satisfactory phytochemical and antioxidant activity. Our *in vitro* digestibility results support the hypothesis of the protective effect of the microencapsulation matrices, allowing a slow release of anthocyanins from the food matrices during simulated gastric digestion and a significant release of the bioactive compounds into the gut. As for textural parameters, the addition of microencapsulated grape juice caused a significant decrease in firmness, cohesiveness and springiness, leading to the destabilization of the gel structure while reducing the attraction forces between the matrix components. The sensorial analysis indicated that the panellists preferred the sample with grape juice the most.
